# NGAL promotes recruitment of tumor infiltrating leukocytes

**DOI:** 10.18632/oncotarget.25625

**Published:** 2018-07-20

**Authors:** Francesco Pacifico, Luna Pisa, Stefano Mellone, Michele Cillo, Alessio Lepore, Antonio Leonardi

**Affiliations:** ^1^ Istituto di Endocrinologia ed Oncologia Sperimentale, CNR, 80131 Naples, Italy; ^2^ Dipartimento di Medicina Molecolare e Biotecnologie Mediche, “Federico II” University of Naples, 80131 Naples, Italy

**Keywords:** NF-κB, NGAL, cancer, chemokines, chemotaxis

## Abstract

We have previously shown that Neutrophil Gelatinase-Associated Lipocalin (NGAL) is strongly expressed in thyroid carcinomas, especially of anaplastic type, where it protects neoplastic cells from serum deprivation-induced apoptosis and enhances tumor invasivity by regulating MMP-9 activity. Here we demonstrate that NGAL-containing conditioned medium from human anaplastic thyroid carcinoma (ATC) cells is able to induce monocyte migration *via* up-regulation of a number of different chemokines. The enhanced chemokines transcription is due to the NGAL-mediated intracellular iron uptake. Very importantly, mice tumor allografts raised from subcutaneous injection of syngeneic colon carcinoma cell lines, expressing high levels of NGAL, show a dense leukocyte infiltrate which strongly decreases in tumor allografts from NGAL-depleted cell injected mice.

Our results indicate that the NGAL promotes leukocytes recruitment in tumor microenvironment through iron-mediated chemokines production.

## INTRODUCTION

The tumor microenvironment plays a pivotal role in the establishment and further development of cancer [1]. It is constituted of a number of different cell types including stromal fibroblasts, endothelial cells and immune cells which synergistically with neoplastic cells deeply contribute to tumor growth and to the multiple stages of tumor progression [2]. Infiltrating immune cells represent one of the most abundant and important components of tumor microenvironment so much so the overwhelming majority of solid tumors shows infiltrates of leukocytes from myeloid and lymphoid origin [3], whose complexity and activation status depend on cancer localization and tumor stage [4, 5]. Tumor infiltrating immune cells induce the synthesis of a number of growth factors, cytokines, chemokines and pro-inflammatory mediators that stimulate proliferation of neoplastic and stromal cells [6]. In addition, tumor infiltrating immune cells secrete different proteolytic enzymes that can alter the structure and function of extracellular matrix (ECM) [7].

The presence of immune cells, other than stromal fibroblasts and endothelial cells, strongly indicates that the inflammatory response plays a strategical role in the onset of cancer. Epidemiological studies have revealed that chronic inflammation predisposes to different forms of cancer including those not causally related to an obvious inflammatory process. The interplay between epithelial and inflammatory cells is thought to be crucial for the genesis and the establishment of carcinomas. One of the main actors in the inflammatory process is NF-κB which, by regulating the expression and the function of different cytokines and chemokines in inflammatory cells, stimulates the growth and its own activity in epithelial cells. Thus, NF-κB establishes a network that, after prolonged time, can lead epithelial cells to undergo malignant transformation [8]. Once activated in neoplastic cells, NF-κB promotes transcription of a number of genes that on one hand contribute to the establishment of its constitutive activation by an autocrine fashion, and on the other hand allows the recruitment of inflammatory and immune cells by a paracrine fashion, thus facilitating the activity of neighbouring tumor cells [9]. Neutrophil Gelatinase-Associated Lipocalin (NGAL) is an iron-binding acute phase protein [10–12] under NF-κB transcriptional regulation [13–15], strongly over-expressed in many human tumors [16–19] including thyroid cancer, where it is able to mediate critical NF-κB functions, such as the resistance to serum withdrawal-induced apoptosis [15] and the metastatic activity of anaplastic thyroid carcinoma cells [20].

Given the pro-inflammatory properties and the de-regulated expression of NGAL in neoplastic cells, we investigated its role in tumor infiltrating immune cells recruitment. Our results indicate that NGAL induced leukocytes recruitment in tumor allografts through iron-mediated up-regulation of a number of chemokines.

## RESULTS

### NGAL promoted monocyte recruitment *in vitro*

**Figure 1:**
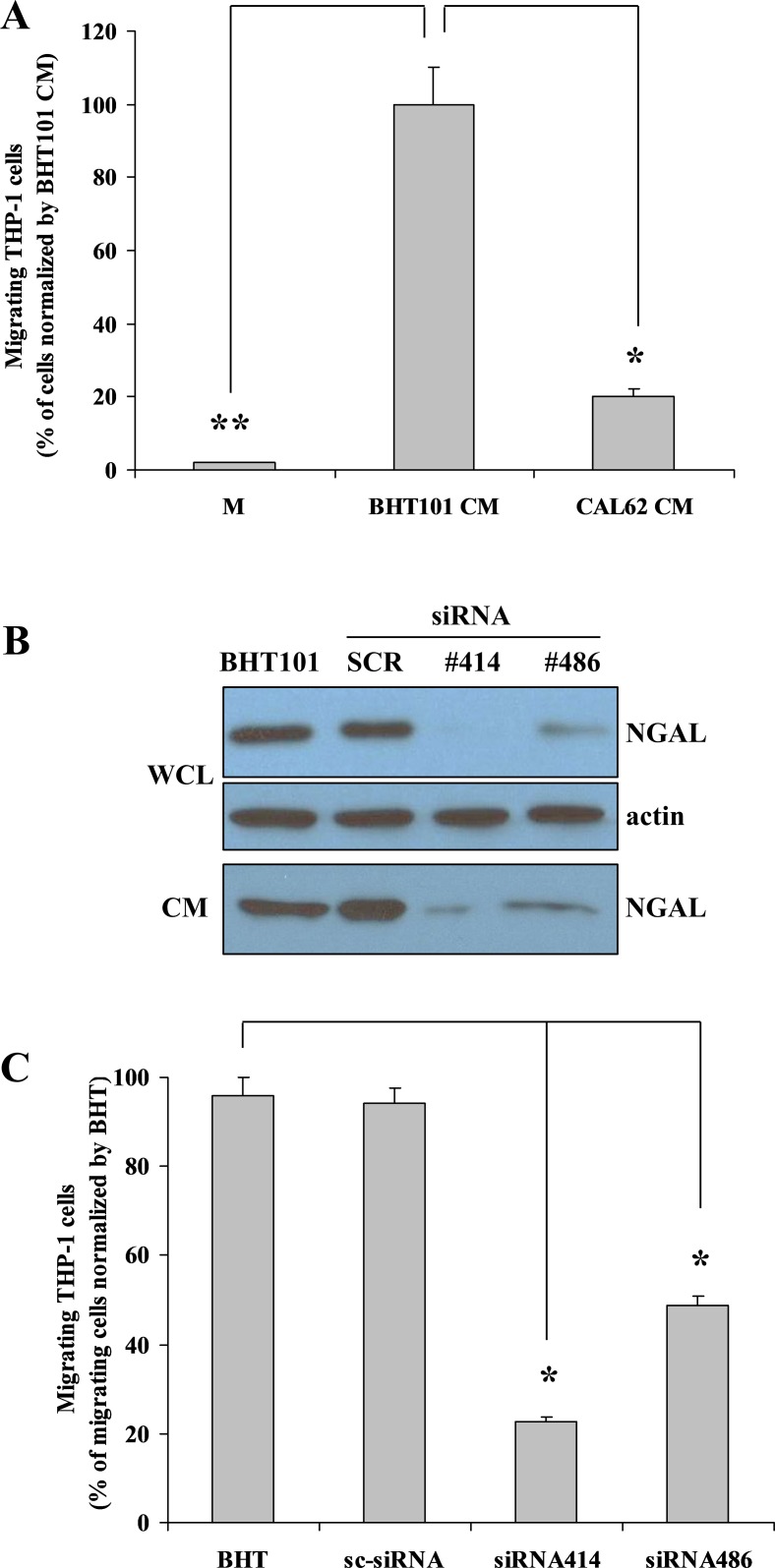
*In vitro* NGAL-mediated leukocytes recruitment Conditioned medium from BHT101 cells promotes THP-1 cells migration compared to medium only and to CAL62 conditioned medium **(A)**. Knocking-down NGAL expression in BHT101 cells **(B)** determines a decreased THP-1 cells chemotaxis compared to parental and scrambled BHT101 cells **(C)**. Data represent mean (± standard deviation) of three technical replicates within a single experiment. Each experiment has been repeated at least three times. Statistical analysis by unpaired Student’s t-test: ^*^, p<0.001. M: unconditioned medium CM: conditioned medium; WCL: whole cell lysate.

To study the potential chemoattractant activity of NGAL, we tested the ability of the conditioned medium from human anaplastic thyroid carcinoma (ATC)-derived BHT101 cells, containing large amounts of NGAL [20], to induce human monocytic THP-1 cells chemotaxis in transwell migration assays. As shown in Figure 1A, BHT101 conditioned medium, in the absence of serum, stimulated THP-1 migration more than 10-fold compared to the medium alone without serum and about 5 fold compared to the conditioned medium from NGAL-negative CAL62 cells. Thus, to validate the hypothesis of NGAL involvement in mediating the chemotactic property of BHT101 conditioned medium, we inhibited its expression in BHT101 cells by infection with the pLenti-siRNA-GFP lentiviral vector containing small interfering RNA (siRNA) sequences (siRNA 414 and siRNA 486) that bind to different regions of NGAL mRNA. BHT101 cells infected with pLenti-siRNA 414 and pLenti-siRNA 486 showed a strong decrease of NGAL expression, both intracellularly and extracellularly, compared to uninfected parental cells and to control scrambled siRNA (Figure 1B). Conditioned media from NGAL proficient and deficient BHT101 cell lines were used to stimulate THP-1 cells chemotaxis in transwell migration assays. As shown in Figure 1C, NGAL-containing culture media from parental and scrambled (sc-siRNA) BHT101 cell lines induced monocytes migration which, instead, was drastically reduced by the depletion of NGAL in conditioned media from BHT101 siRNA414 and siRNA486 cells.

These results suggest that NGAL could act as a monocyte chemoattractant protein in the extracellular medium of human ATC cell lines.

### NGAL up-regulated chemokines expression in BHT101 cells by autocrine fashion

Since it has never been demonstrated an intrinsic chemotactic property of NGAL, we hypothesized that its ability to recruit THP-1 cells could occur through the up-regulation of chemokines expression in BHT101 cells *via* autocrine stimulation. To verify this hypothesis, parental, scrambled and NGAL-depleted BHT101 cell lines were cultured in medium without serum and the expression of a set of human chemokines was analysed by qRT-PCR. We analysed expression of seven inflammatory chemokines involved in trafficking of different cell populations into the tumor microenvironment [21, 22].

**Figure 2:**
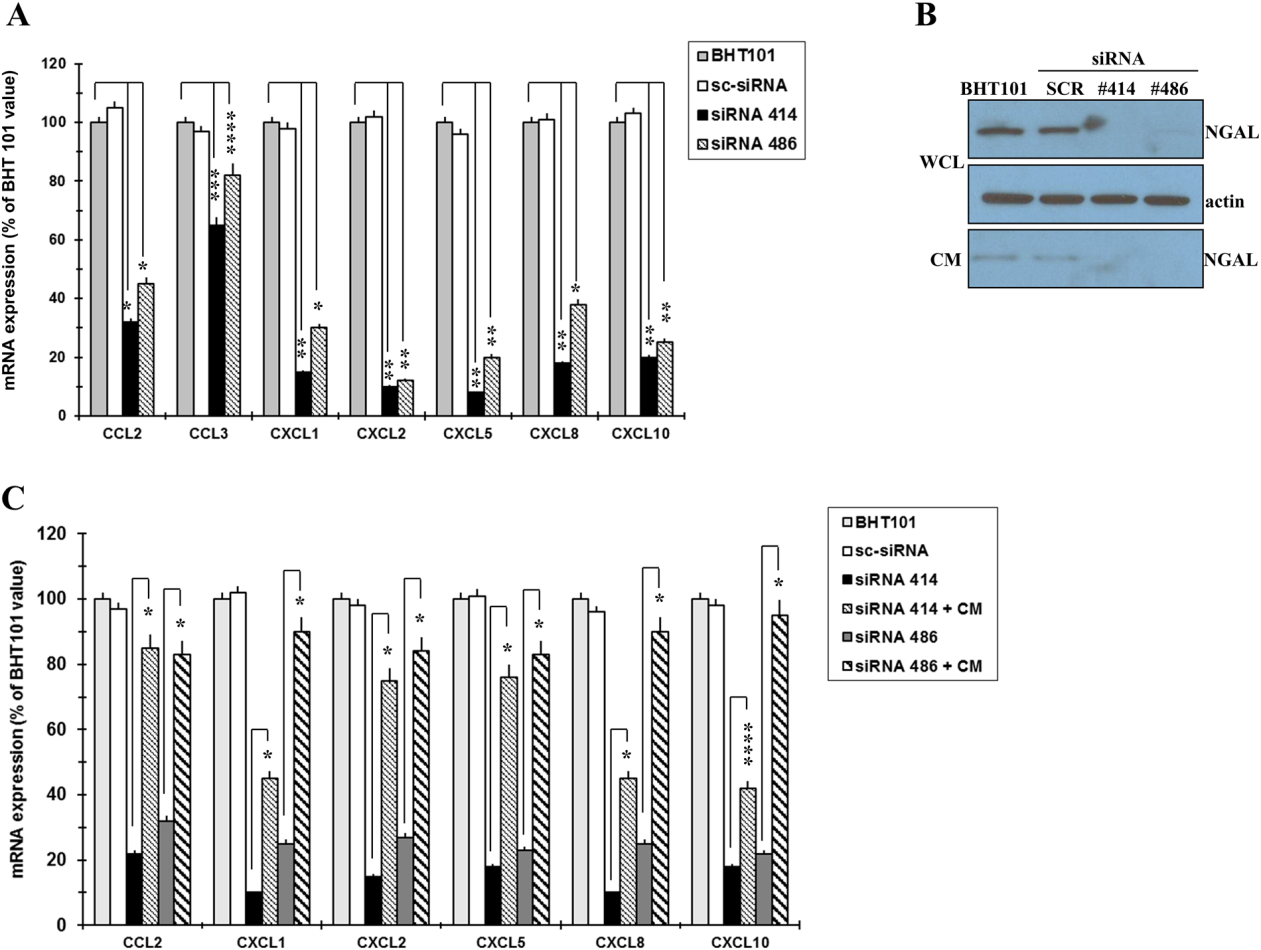
NGAL influences chemokines expression in BHT101 cells qRT-PCR analysis showing chemokines expression in NGAL-depleted BHT101 cells compared to parental and scrambled counterpart **(A)**. Western blot showing that NGAL is rapidly secreted from BHT101 cell lines after 1h of incubation in serum-free medium (WCL: whole cell lysate; CM: conditioned media) **(B)**. Chemokines expression as assessed by qRT-PCR in NGAL-depleted BHT101 cells after the addition of NGAL-containing conditioned medium from parental BHT101 cells **(C)**. Data represent mean (± standard deviation) of three technical replicates within a single experiment. Each experiment has been repeated at least three times. Statistical analysis by unpaired Student’s t-test: ^*^, p<0.001; ^**^, p<0.0001; ^***^, p<0.005; ^****^, p<0.01.

The depletion of NGAL in BHT101 cells determined a strong mRNA decrease of all the chemokines analysed, even so at different extent, compared to parental and scrambled BHT cells (Figure 2A). To confirm the role of NGAL in the regulation of chemokines expression, BHT NGAL- depleted cells were cultured in medium without serum for 1 hour at 37°C and, then, incubated with NGAL-containing conditioned media from parental or scrambled BHT cells. The addition of exogenous NGAL to BHT101 siRNA 414 and 486 cells restored chemokines expression, even though at different extent (Figure 2C).

These data indicate that ATC cells-secreted NGAL promotes monocyte chemotaxis by up-regulating a number of chemokines.

### Chemokines up-regulation depended on NGAL-mediated iron uptake

**Figure 3:**
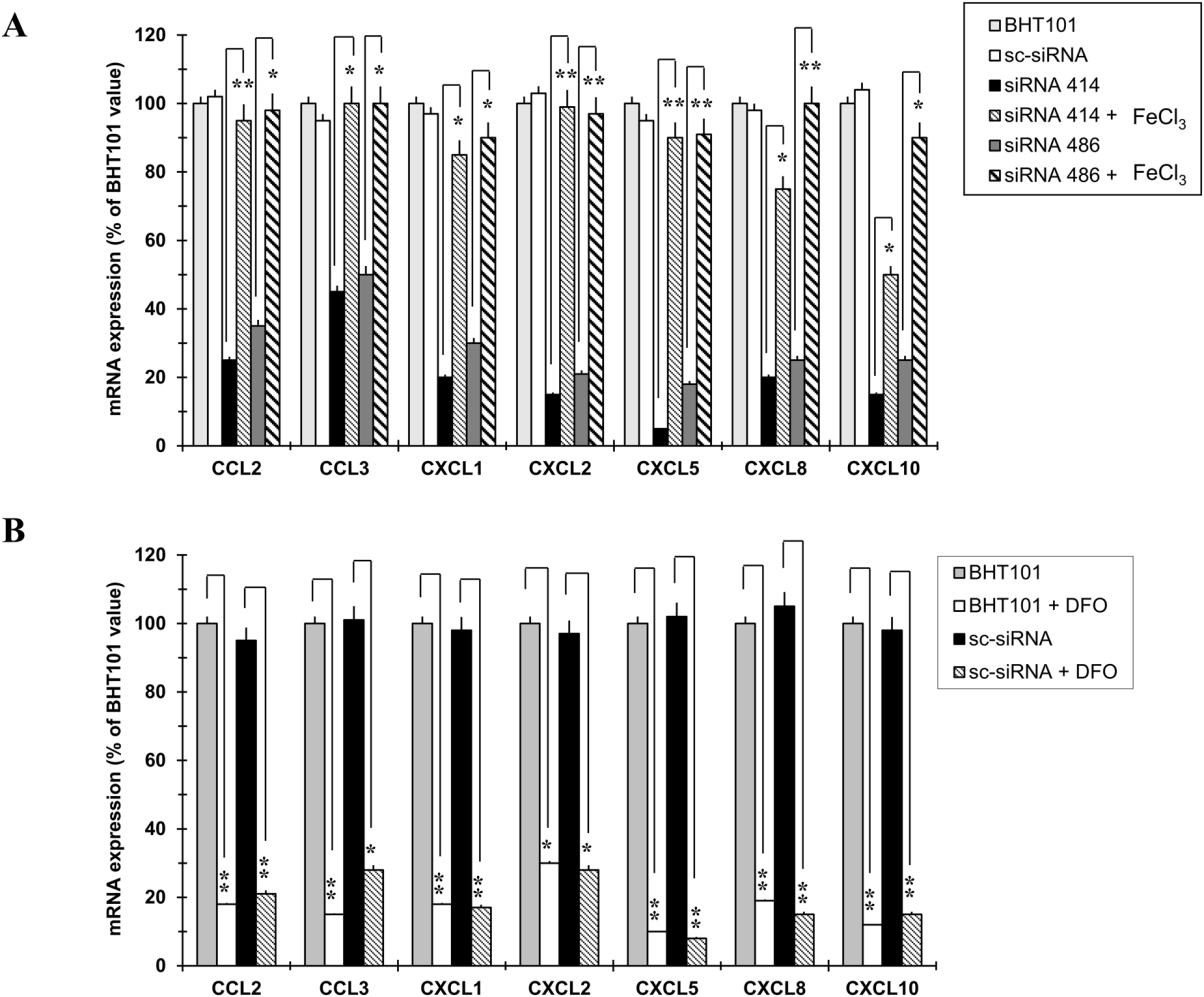
Iron mimics NGAL effects on chemokines expression in BHT101 cell lines Analysis of chemokines expression by qRT-PCR after treatment of NGAL-depleted BHT101 cell lines with FeCl_3_ **(A)**, and after the addition of the iron chelator DFO to the culture medium of parental and scrambled BHT101 cells **(B)**. Data represent mean (± standard deviation) of three technical replicates within a single experiment. Each experiment has been repeated at least three times. Statistical analysis by unpaired Student’s t-test: ^*^, p<0.0001; ^**^, p<0.00001

Since one of the main function of NGAL is to transport iron inside cells and given that iron exerts a pivotal role in the biology of cancer cells, we hypothesized that NGAL could sustain chemokines transcription through the regulation of intracellular iron concentration. Therefore, BHT101 NGAL-depleted cell lines were starved in medium only and, after 1 hour, they were incubated in FeCl_3_-containing medium, in order to analyse if the addition of exogenous iron could restore chemokines expression. As shown in Figure 3A, the presence of 50 μM FeCl_3_ in the culture medium of BHT101 siRNA 414 and 486 cells re-activated the expression of all seven chemokines (normal and bolded striped bars) at an extent similar to that of parental and scrambled BHT101 cells (light grey and white bars). On the contrary, the use of the iron chelator deferoxamine (DFO) determined a strong down-regulation of chemokines expression in parental and scrambled BHT101 cells (Figure 3B, white and striped bars) if compared to that of untreated cells (Figure 3B, grey and black bars).

**Figure 4:**
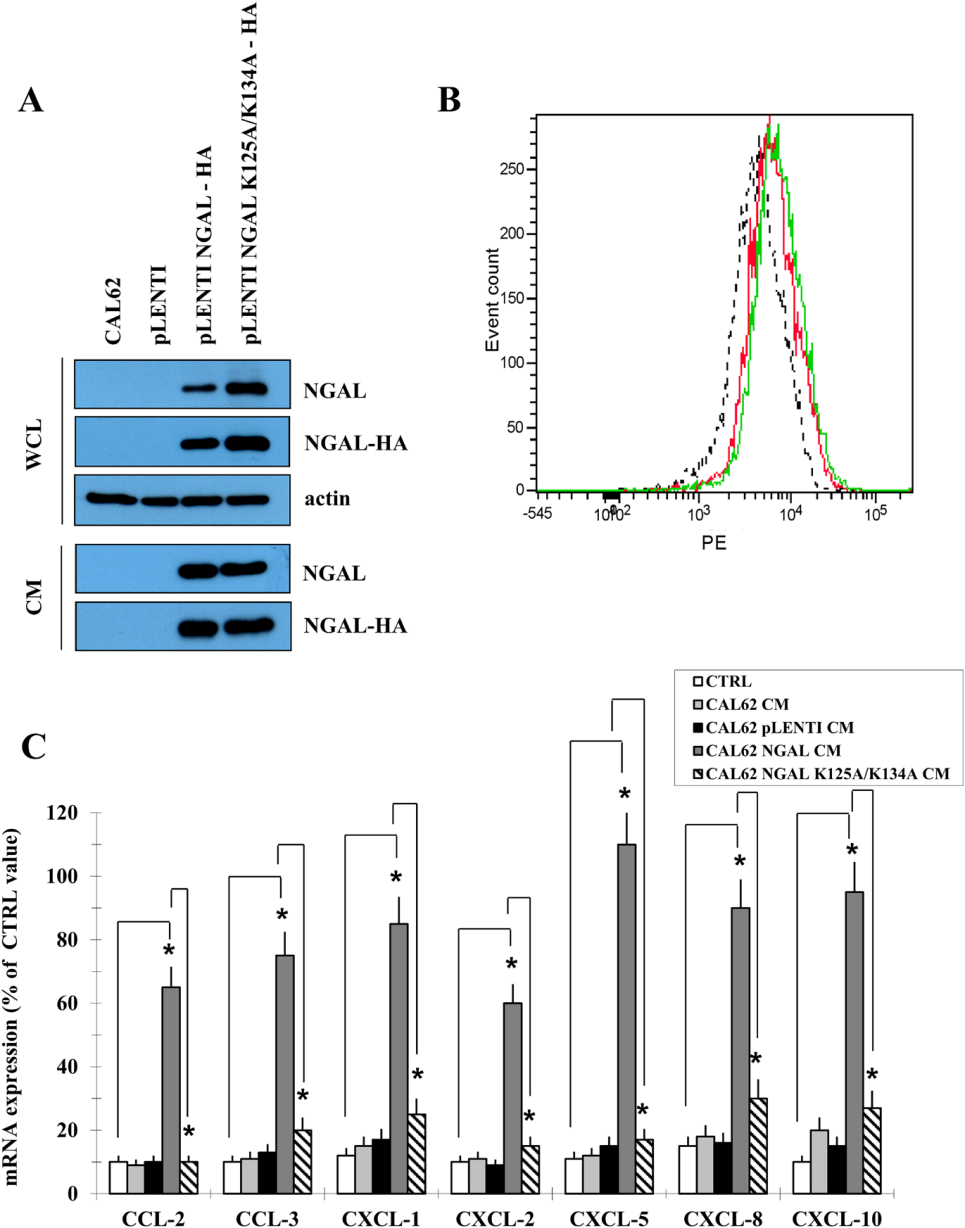
A mutant form of NGAL unable to bind siderophores did not restore chemokines expression Western blot of wild type NGAL and the mutant form NGAL K125A-K134A fused in frame with HA tag exogenously expressed in CAL62 cells **(A)**. FACS analysis of wild type (red) and mutant (green) NGAL internalized by BHT101 siRNA 486 cells challenged with conditioned media from infected CAL62 cells. Fluorescence of internalized proteins was compared to that obtained with conditioned medium from empty vector infected CAL62 cells (dotted line) **(B)**. Conditioned media were then added to the BHT 101 siRNA 486, and after 24 hours the expression of chemokines was assessed by RT-PCR **(C)**. Data represent mean (± standard deviation) of three technical replicates within a single experiment. Each experiment has been repeated at least three times. Statistical analysis by unpaired Student’s t-test: ^*^, p<0.0001. CM: conditioned medium; WCL: whole cell lysate.

To further demonstrate that the iron-mediated chemokines up-regulation was due to the NGAL-dependent intracellular iron delivery, a mutant form of NGAL carrying two aminoacid substitutions (K125A and K134A) unable to bind siderophores and therefore iron [23], was ectopically expressed in the NGAL-deficient anaplastic thyroid carcinoma cell line CAL62 (Figure 4A). BHT101 siRNA 486 cells were incubated with non-conditioned medium (CTRL), and with conditioned media from parental (CAL62 CM) and mock (CAL62 pLENTI-CM), as well as from wild type (CAL62 NGAL CM) and mutant NGAL (CAL62 NGAL K125A/K134A CM) infected CAL62 cells, and chemokines mRNA levels were evaluated. As shown in Figure 4C, wild type NGAL restored chemokines expression in BHT101 siRNA 486 cells (Figure 4C dark grey vs. white bars), while the iron-deficient NGAL mutant K125A/K134A did not (Figure 4C striped vs. white bars). Of note, both NGAL and NGAL K125A/K134A were internalized by BHT101 siRNA 486 cells (Figure 4B).

These findings demonstrate that iron influences chemokines expression in anaplastic thyroid carcinoma cells and suggest that NGAL-mediated chemokines up-regulation is dependent on its iron-binding properties.

### Decreased NGAL secretion from neoplastic cells impaired immune cells recruitment in tumor microenvironment

**Figure 5:**
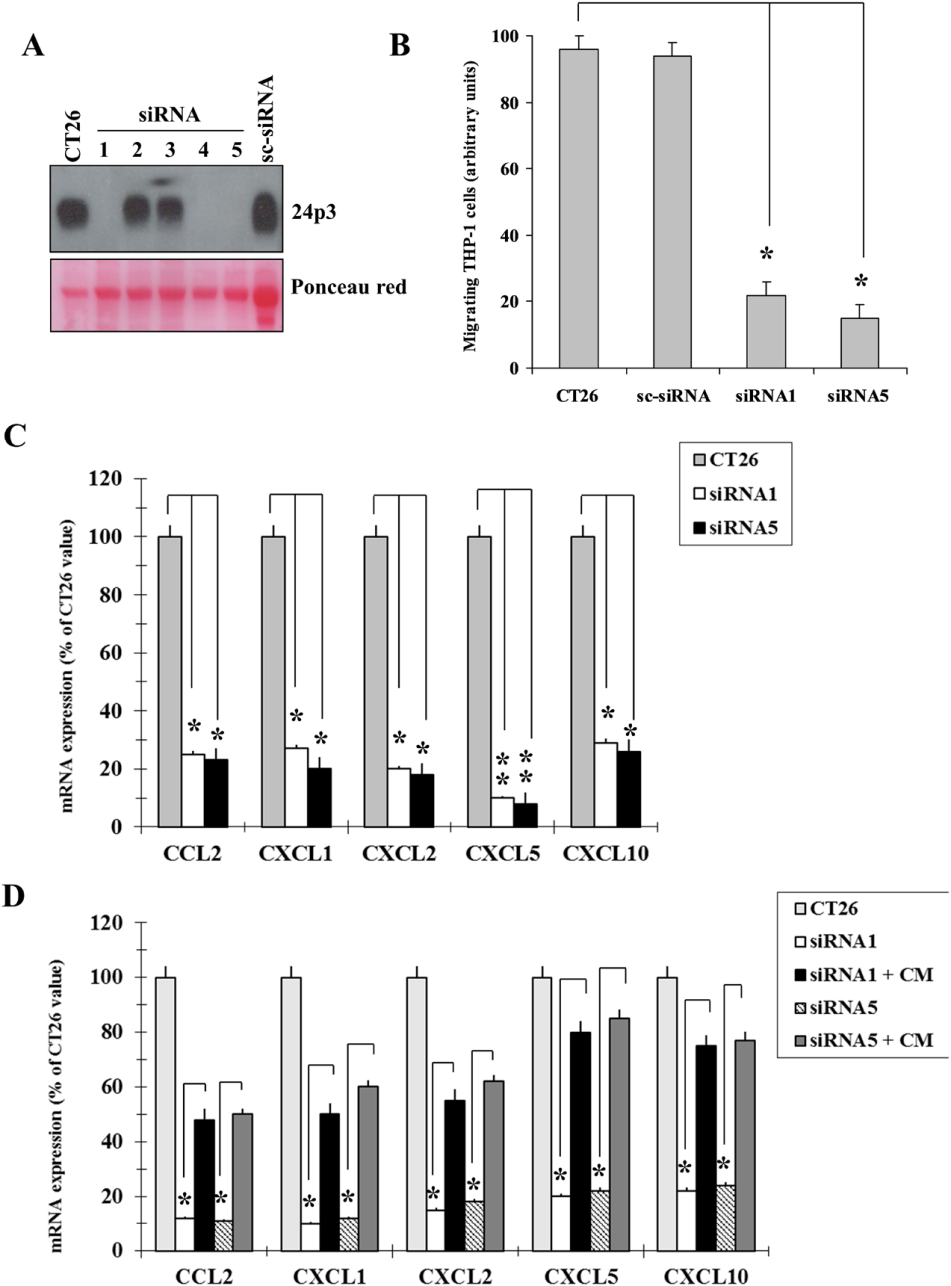
Mouse 24p3 recapitulates human NGAL behaviour Western blot showing expression of 24p3 in mouse colon carcinoma CT26 cells and knocked-down clones **(A)**. Transwell migration assay showing THP-1 cells migration driven by conditioned media from CT26 knocked-down clones **(B)**. Chemokines expression in CT26 knocked-down clones assessed by qRT-PCR analysis **(C)**. Conditioned media from parental CT26 cells were added to the knocked-down clones, and after 24 hours chemokines expression was assessed by qRT-PCR **(D)**. Data represent mean (± standard deviation) of three technical replicates within a single experiment. Each experiment has been repeated at least three times. Statistical analysis by unpaired Student’s t-test: ^*^, p<0.0001; ^**^, p<0.00001. CM: conditioned medium.

**Figure 6:**
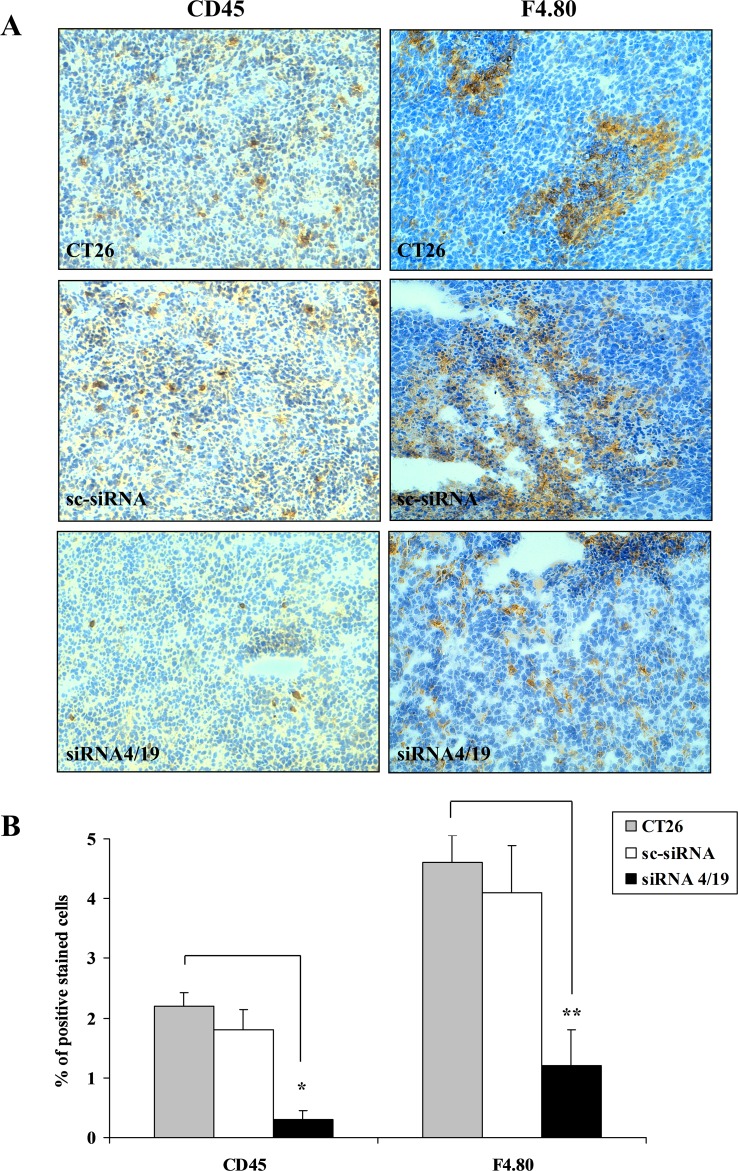

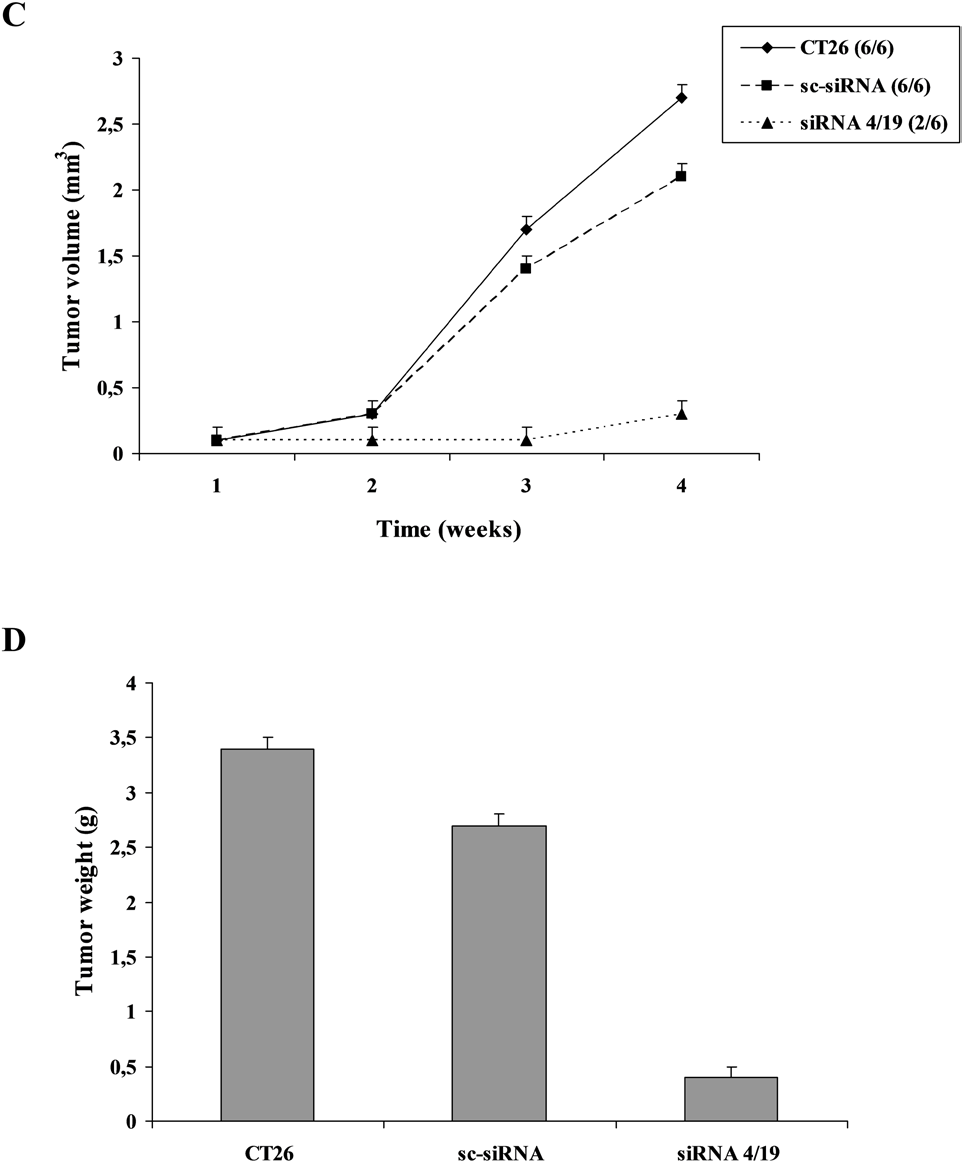
*In vivo* analysis of leukocytes infiltrate in tumor microenvironment of mice xenografts Immunohistochemistry **(A)** of tumors generated by CT26 cell lines injected into syngeneic mice stained with anti-CD45 (left panel) for lymphocytes detection and with anti-F4/80 (right panel) for macrophages detection. CD45 positive lymphocytes and F4/80 positive macrophages are detected as brown stained cells on a background of blue unstained tumor cells. Magnification: 10x. **(B)** Quantification of brown stained cells, from six mice allografts for each experimental group analyzed by ImageJ software (NIH, Bethesda MD, USA). Results are mean ±SD. Ultrasound analysis of tumor mass in each group of 6 mice injected with different CT26 cell lines **(C)**. Tumor weights 4 weeks after cells inoculation **(D)**. Statistical analysis by unpaired Student’s t-test ^*^, p<0,0001; ^**^, p<0,001

Given the chemoattractant activity of NGAL *in vitro*, we investigated this ability *in vivo*. Since BHT101 cells are of human origin, we chose to perform the *in vivo* experiments with the mouse colon carcinoma CT26 cell line [24] that expressed large amounts of 24p3 (the mouse homologous of human NGAL) (Figure 5A). To this end, NGAL expression was knocked down in CT26 cells by stable transfection of pcRNAi plasmid containing siRNA sequences to block 24p3 expression. At least two stable clones, CT26 siRNA1 and siRNA5, showed an almost undetectable level of NGAL expression in their conditioned media if compared to parental and scrambled CT26 cells (Figure 5A). Similarly to BHT101 NGAL-depleted cells, depletion of 24p3 in the extracellular milieu of CT26 24p3-depleted cells determined a reduced ability to induce THP-1 chemotaxis in transwell migration assays (Figure 5B), and a decreased chemokines expression (Figure 5C) that was restored after challenging with 24p3-containing conditioned medium from parental CT26 cells (Figure 5D). Subcutaneous injection of parental, scrambled and 24p3-depleted cells into the flanks of syngeneic mice gave rise to tumors of different sizes (Figure 6C and 6D). Immunohistochemistry of tumor masses arising from parental and scrambled CT26 cells showed a dense infiltrate of lymphocytes, stained with anti-CD45 antibodies (Figure 6A, left panel, and Figure 6B), and macrophages, stained with anti-F4-80 antibodies (Figure 6A, right panel, and Figure 6B). However, the same antibodies used for the leukocytes staining in tumor allografts from CT26 NGAL/24p3-depleted cells revealed an impressive reduced number of infiltrating immune cells (Figure 6A and 6B).

These results indicate that NGAL could act as a regulator of immune cells recruitment in tumor microenvironment through its ability to modulate chemokines expression of neoplastic cells.

## DISCUSSION

In the present paper we demonstrate that NGAL secreted by neoplastic cells supports immune cells chemotaxis in tumor microenvironment. Culture media from NGAL-depleted human anaplastic thyroid carcinoma cells and from mouse colon carcinoma cells show a decreased ability to promote monocyte migration *in vitro* because of a drastic reduction of chemokines expression. Our results show that monocyte chemotaxis is not completely abrogated very likely for at least two reasons: i) the residual NGAL expression in knocked-down cells, might be still sufficient to stimulate a slighter chemokine synthesis; ii) other chemokines, besides those analyzed in the present work, could contribute to monocyte recruitment in a NGAL-independent fashion. Nevertheless, the contribution of NGAL to fuel immune infiltrate in tumor microenvironment is crucial given that neoplasms isolated from mice injected with NGAL-depleted cells show a strong decrease of infiltrating lymphocytes and macrophages compared to neoplasms from mice injected with NGAL-proficient cells. Interestingly, only 30% of mice injected with NGAL-deficient cells develops tumors, that were smaller and arose later than allografts derived from the injection of NGAL-proficient cells.

The pivotal role played by NGAL in the regulation of chemotaxis driven by neoplastic cells is proven by the evidence that the addition of NGAL-containing conditioned media from control cancer cells restores chemokines expression. The ability of NGAL to act as a chemokine inducer has been studied in models of mouse neuroinflammation in which NGAL promote astrocytes migration mainly through CXCL-10 up-regulation in different central nervous system cells [25–27]. Moreover, NGAL has been demonstrated to mediate chemokine expression and granulocyte infiltration during ischemia and reperfusion in a mouse model of heart transplantation [28] and to induce cytokine production to enhance endometrial carcinoma cell survival and migration [29]. Here we demonstrate that NGAL synthesized by cancer cells promotes leukocytes chemotaxis to tumor microenvironment as a result of a sustained chemokine expression by neoplastic cells. By this way, NGAL could contribute to thyroid cancer progression not only by its anti-apoptotic [15] and pro-metastatic [20] activities, but also by its ability to support tumor immune infiltrate. We did not investigate the molecular pathway by which iron-loaded NGAL activates chemokines transcription, however it is possible to speculate that chemokines promoter specific transcription factors are activated by iron-loaded NGAL following NGAL receptor (NGALR)-mediated uptake from cancer cells conditioned media. Interestingly, NGAL-proficient and NGAL-deficient ATC cells show no differences in NGALR expression (data not shown) suggesting that chemokines decrease is due to reduced NGAL availability in extracellular milieu rather than to NGALR down-regulation.

Our findings also point out the role of iron in the NGAL-mediated chemokine up-regulation: i) NGAL-depleted ATC cells challenged with a mutated form of NGAL unable to bind siderophores, did not rescue chemokine expression; ii) the addition of the iron chelator DFO in cell culture medium determines a strong decrease of chemokine expression in ATC cells; iii) the presence of exogenous FeCl_3_ restores chemokines expression. Iron, delivered inside the cells by different carriers, such as transferrin, is crucial for cancer cells proliferation, survival and metastatic spread [30]. Different studies report that malignant cells show over-expression of transferrin receptor, ferritin and the iron-regulatory protein-2 (IRP-2), which play a role in the intracellular iron delivery and storage, and, on the contrary, down-regulation of ferroportin that mediates iron efflux from cells [31–35]. Tumor microenvironment seems to be the major source of iron for cancer cells given the ability of resident macrophages to phagocytose erythrocytes from tumor blood vessels and to release iron to neoplastic cells to support tumor progression [30]. Besides transferrin, it has been demonstrated that iron could be delivered into malignant cells by different alternative routes, for example following the binding to NGAL [16–18] or to siderophores [30]. In a previous work we reported that NGAL-deficient ATC cells have a lower iron content than NGAL-proficient ATC cells and undergo apoptosis following serum deprivation at higher rate than NGAL-proficient ATC cells [15]. Challenging NGAL-depleted cells with iron-loaded NGAL rescues them from serum deprivation-induced apoptosis [15]. In mice colon carcinomas NGAL-deficient tumors have reduced iron content and are smaller than tumors developed in NGAL expressing mice [36]. Therefore, it is conceivable that a transferrin-independent way of iron transport has been developed by malignant cells to meet their increased iron demand and that NGAL is one of the main candidates to exert this function.

In conclusion we propose a novel mechanism by which NGAL contributes to tumor progression highlighting the yet undiscovered role of NGAL in the regulation of tumor microenvironment homeostasis. NGAL in addition to support tumor growth by providing iron to cancer cells in an autocrine fashion, can also influence tumor microenvironment by increasing transcription of chemokines ultimately leading to macrophage recruitment.

## MATERIALS AND METHODS

### Cell culture and biological reagents

The human anaplastic thyroid carcinoma BHT101 and CAL62 cell lines were kindly provided by Prof. M. Santoro, “Federico II” University of Naples, Italy, and the murine colon carcinoma CT26 cell line by Prof. G. Monteleone, “Tor Vergata” University of Rome, Italy. BHT101 cells were grown in Dulbecco's modified Eagle's medium (DMEM) (Sigma, St. Louis, MO, USA) supplemented with 20% foetal bovine serum (Sigma). CAL62 cells were grown in Dulbecco's modified Eagle's medium (DMEM) (Sigma, St. Louis, MO, USA) supplemented with 10% foetal bovine serum (Sigma). CT26 and human monocytic THP-1 cell lines were grown in RPMI 1640 medium (Sigma) supplemented with 10% foetal bovine serum (Sigma).

To knock-down NGAL/24p3 expression, BHT101 cells were infected with pLenti-siRNA-GFP lentiviral vector (Applied Biological Materials Inc., Richmond BC, Canada) containing double-stranded oligonucleotides sequences derived from the human NGAL: siRNA 414, 5′-TGCTATGGTGTTCTTCAAGAAAGTTTCTC-3′ and siRNA 486, 5′-GGAGCTGACTTCGGAACTAAAGGAGAACT-3′. The scrambled pLenti-siRNA-GFP (sc-siRNA, 5′-GGGTGAACTCACGTCAGAA-3′) was used as a negative control. CT26 cells were transfected with pcRNAi vector [37] containing double-stranded oligonucleotides sequences derived from the murine 24p3 in forward and reverse orientation, separated by a 7-bp spacer region (caagaga) to allow the formation of the hairpin structure in the expressed siRNAs: siRNA 4, 5′-GGCAGCTTTACGATGTACAcaagagaTGTACATCGTAAAGCTGCC-3′ (sense strand) and 5′-GGCAGCTTTACGATGTACAtctcttgTGTACATCGTAAAGCTGCC-3′ (antisense strand). The scrambled siRNA (sc-siRNA) was: 5′-CATTTGTTCCAAGCTCCAGcaagagaCTGGAGCTTGGAACAAATG-3′ (sense strand) and 5′–CATTTGTTCCAAGCTCCAGtctcttgCTGGAGCTTGGAACAAATG-3′ (antisense strand).

To over-express wild type NGAL and the mutant form NGAL K125A-K134A, CAL62 cells were infected with pLenti-CMV-GFP-2A-Puro lentiviral vector (ABM, Richmond, BC, Canada) containing the cDNA coding for human wild type NGAL, or for a mutant form of NGAL, in which two lysine residues in positions 125 and 134 have been replaced with two alanine residues (K125A-K134A), fused in frame with HA tag.

Anti-actin (sc-8432) and anti-HA (sc-805) were purchased from Santa Cruz Biotechnology, Inc. (Santa Cruz, CA), anti-NGAL (AF1757) and anti-24p3 (AF1857) were from R&D Systems (Minneapolis, MN, USA), anti-F4/80 (MCA497R) was from AbD Serotec (Oxford, UK), anti-CD45 (ab10558) was from Abcam (Cambridge, MA, USA).

### Western blots

For Western blots, 20 μg of total proteins from cell lysates or supernatants were analyzed by 10% SDS-PAGE and blotted onto nitrocellulose membrane (Schleicher & Schuell, Whatman GmbH). Filters were blocked for 1 h 30 min at room temperature with 5% nonfat dry milk in TBST buffer (10mM Tris-HCl pH 8), 0.1% Tween-20, 150 mM NaCl) and incubated with 1:2000 dilution of anti-HA, anti-NGAL, anti-24p3 or anti-actin antibodies for 1 h 30 min. After TBST washing, blots were incubated for 1 h with horseradish peroxidase-conjugated secondary antibodies (GE Healthcare, UK) diluted 1:5000 in TBST buffer and then revealed by ECL (GE Healthcare, UK).

### NGAL internalization assay

To detect internalization of NGAL and NGAL K125A-K134A, the conditioned media isolated from HA-NGAL- and HA-NGAL K125A-K134A-infected Cal62 cells, were added to BHT101 siRNA 486 cells for 24 hours. Then, cells were collected, permeabilized with Perm buffer (BD Biosciences), incubated with anti-HA and stained by using PE-conjugated secondary antibody. Fluorescent cells were analysed by FACS Canto II (BD Biosciences).

### Transwell migration assays

1 × 10^6^ THP-1 cells in 100 μl of serum-free Opti-MEM I (Invitrogen, Carlsbad, CA, USA) were placed on the polycarbonate membranes (5-μm pore size) in the upper compartment of the transwells (6.5 mm, Costar, Cambridge, MA, USA), while 600 μl of Opti-MEM I without serum or of conditioned media from human or mouse cell lines, grown for 48 h in Opti-MEM I without serum, were added to the lower compartment. The plates were incubated at 37°C in a 5% CO_2_ atmosphere saturated with H_2_O for 1 h. At the end of incubation, the cells at the upper side of the polycarbonate filter were mechanically removed. Cells that had migrated to the lower compartment through the filter were counted with a Neubauer chamber. The error bars represent technical replicates within a single experiment. Each experiment has been repeated at least three times.

### Immunohistochemistry of tumor xenografts

The animal protocol used in this work was approved by the animal use and ethic committee of the Federico II University of Naples (Protocol 587/2015). Three groups of six mice (6-week-BALB/c, Charles River, Lecco, Italy) for each tumor cell line were used to analyze *in vivo* tumorigenicity of different CT26 cell lines. 2 × 10^7^ cells were injected s. c. on a flank of each mouse. Tumor growth was monitored by ultrasound analysis every week along four weeks. At the end of fourth week, mice were killed and tumors were excised, weighted, rinsed with PBS, fixed in 4% buffered neutral formalin and embedded in paraffin. Then, 5-6 μm thick paraffin sections were deparaffinized and placed in a solution of absolute methanol and 0.3% hydrogen peroxide for 30 min, then washed in PBS before immunoperoxidase staining. Slides were then incubated overnight at 4°C in a humidified chamber with antibody anti-CD45 or anti-F4/80 diluted 1:100 in PBS and subsequently incubated, first with biotinylated goat anti-rabbit or rabbit anti-rat IgG for 20 min (Vectostain ABC kits, Vector Laboratories), and then with pre-mixed reagent ABC (Vector) for 20 min. The immunostaining was performed by incubating slides in diaminobenzidine (DAB-DAKO) solution containing 0.06 mM DAB and 2 mM hydrogen peroxide in 0.05% PBS pH 7.6 for 5 min, and after chromogen development, slides were washed, dehydrated with alcohol and xylene, and mounted with coverslips using a permanent mounting medium (Permount).

**Table:** 

HUMAN	MOUSE
GAPDH	GAPDH
Fwd 5′-ATGGTGAAGGTCGGTGTGAAC-3′	Fwd 5′-ATGGTGAAGGTCGGTGTGAAC-3′
Rev 5′-CCATGTAGTTGAGGTCAATGAAG-3′	Rev 5′-CCATGTAGTTGAGGTCAATGAAG-3′
**CCL2**	**CCL2**
Fwd 5′-TGTCCCAAAGAAGCTGTGATC-3′	Fwd 5′-AGCTGTAGTTTTTGTCACCAAGC-3′
Rev 5′-ATTCTTGGGTTGTGGAGTGAG-3′	Rev 5′-GTGCTGAAGACCTTAGGGCA-3′
**CCL3**	**CXCL1**
Fwd 5′-CAGCTACACCTCCCGGCA-3′	Fwd 5′-CATGGCTGGGATTCACCTCAAGA-3′
Rev 5′-TCGCTTGGTTAGGAAGATGAC-3′	Rev 5′-GGAGCTTCAGGGTCAAGGCAAG-3′
**CXCL1**	**CXCL2**
Fwd 5′-CCTCCCTTCTGGTCAGTTG-3′	Fwd 5′-CCCAGACAGAAGTCATAGCCA-3′
Rev 5′-AACCGAAGTCATAGCCACAC-3′	Rev 5′-CAGGTACGATCCAGGCTTCC-3′
**CXCL2**	**CXCL5**
Fwd 5′-AACCGAAGTCATAGCCACAC-3′	Fwd 5′-AGAAGGAGGTCTGTCTGGATCCA-3′
Rev 5′-CTTCTGGTCAGTTGGATTTGC-3′	Rev 5′-CGACTCCATTCCGCTTAGCTTTC-3′
**CXCL5**	**CXCL10**
Fwd 5′-TCTGCAAGTGTTCGCCATAG-3′	Fwd 5′-CCACGTGTTGAGATCATTGCC-3′
Rev 5′-CAGTTTTCCTTGTTTCCACCG-3′	Rev 5′-GAGGCTCTCTGCTGTCCATC-3′
**CXCL8**	**24p3**
Fwd 5′-ATACTCCAAACCTTTCCACCC-3′	Fwd 5′-ACAGAGCTACAATGTGCAAGTG-3′
Rev 5′-TCTGCACCCAGTTTTCCTTG-3′	Rev 5′-CAGCTCCTTGGTTCTTCCATACA-3′
**CXCL10**	
Fwd 5′-TTAAGGGTTACCTGGGTTGCC-3′	
Rev 5′-TCTTGGTTCTCAGCTTGGGGC-3′	
**NGAL**	
Fwd 5′-TCCCTGTCCCAATCGACCAGTGT-3′	
Rev 5′-GAGCAGCTGCATGGGTGGCA-3′	

### RNA extraction and mRNA quantification by real-time RT–PCR

High quality total RNA preparations from cell lines were carried out by TRIZOL according to manufacturer's instructions (Thermo Fisher Scientific, Waltham MA, USA). To avoid the risk of DNA contamination in PCR assays, all RNA samples were treated with RNase free-DNase I (Promega, Fitchburg WI, USA) according to manufacturer's protocol. To analyze mRNA expression, real-time reverse transcription–PCR was carried out with complementary DNAs reverse-transcribed from total RNA by using Transcription First Strand cDNA Synthesis kit and LightCycler 480 Probe Master Mix (Roche, Indianapolis, IN, USA), according to manufacturers’ procedure. Quantitative analysis was performed by LightCycler480 software (Roche) on the basis of the following protocol: denaturation step at 95°C 10 min for 1 cycle, amplification steps at 95°C 10 sec, 60°C 10 sec, 72°C 8 sec for 40 cycles. The *gapdh* gene was used as internal reference gene. Data were calculated by ΔCt method (2^−ΔΔC^^t^). The primers used were:

## Abbreviations

NF-κB: Nuclear Factor-kappa B
CCL and CXCL: Chemokine Ligand
NGAL: Neutrophil Gelatinase-Associated Lipocalin
ATC: anaplastic thyroid carcinoma

## References

[1] Whiteside TL (2008). The tumor microenvironment and its role in promoting tumor growth. Oncogene.

[2] Quail DF, Joyce JA (2013). Microenvironmental regulation of tumor progression and metastasis. Nat Med.

[3] Tlsty TD, Coussens LM (2006). Tumor stroma and regulation of cancer development. Annu Rev Pathol.

[4] Mantovani A, Allavena P, Sica A, Balkwill F (2008). Cancer-related inflammation. Nature.

[5] Ruffell B, Au A, Rugo HS, Esserman LJ, Hwang ES, Coussens LM (2012). Leukocyte composition of human breast cancer. Proc Natl Acad Sci U S A.

[6] Balkwill F, Charles KA, Mantovani A (2005). Smoldering and polarized inflammation in the initiation and promotion of malignant disease. Cancer Cell.

[7] Lu P, Takai K, Weaver VM, Werb Z (2011). Extracellular matrix degradation and remodeling in development and disease. Cold Spring Harb Perspect Biol.

[8] Pacifico F, Leonardi A (2006). NF-kappaB in solid tumors. Biochem Pharmacol.

[9] Ben-Neriah Y, Karin M (2011). Inflammation meets cancer, with NF-κB as the matchmaker. Nat Immunol.

[10] Goetz DH, Holmes MA, Borregaard N, Bluhm ME, Raymond KN, Strong RK (2002). The neutrophil lipocalin NGAL is a bacteriostatic agent that interferes with siderophore-mediated iron acquisition. Mol Cell.

[11] Flo TH, Smith KD, Sato S, Rodriguez DJ, Holmes MA, Strong RK, Akira S, Aderem A (2004). Lipocalin 2 mediates an innate immune response to bacterial infection by sequestrating iron. Nature.

[12] Nilsen-Hamilton M, Liu Q, Ryon J, Bendickson L, Lepont P, Chang Q (2003). Tissue involution and the acute phase response. Ann N Y Acad Sci.

[13] Cowland JB, Sørensen OE, Sehested M, Borregaard N (2003). Neutrophil gelatinase-associated lipocalin is up-regulated in human epithelial cells by IL-1 beta, but not by TNF-alpha. J Immunol.

[14] Cowland JB, Muta T, Borregaard N (2006). IL-1beta-specific up-regulation of neutrophil gelatinase-associated lipocalin is controlled by IkappaB-zeta. J Immunol.

[15] Iannetti A, Pacifico F, Acquaviva R, Lavorgna A, Crescenzi E, Vascotto C, Tell G, Salzano AM, Scaloni A, Vuttariello E, Chiappetta G, Formisano S, Leonardi A (2008). The neutrophil gelatinase-associated lipocalin (NGAL), a NF-kappaB-regulated gene, is a survival factor for thyroid neoplastic cells. Proc Natl Acad Sci U S A.

[16] Bolignano D, Donato V, Lacquaniti A, Fazio MR, Bono C, Coppolino G, Buemi M (2010). Neutrophil gelatinase-associated lipocalin (NGAL) in human neoplasias: a new protein enters the scene. Cancer Lett.

[17] Lippi G, Meschi T, Nouvenne A, Mattiuzzi C, Borghi L (2014). Neutrophil gelatinase-associated lipocalin in cancer. Adv Clin Chem.

[18] Chakraborty S, Kaur S, Guha S, Batra SK (2012). The multifaceted roles of neutrophil gelatinase associated lipocalin (NGAL) in inflammation and cancer. Biochim Biophys Acta.

[19] Rodvold JJ, Mahadevan NR, Zanetti M (2012). Lipocalin 2 in cancer: when good immunity goes bad. Cancer Lett.

[20] Volpe V, Raia Z, Sanguigno L, Somma D, Mastrovito P, Moscato F, Mellone S, Leonardi A, Pacifico F (2013). NGAL controls the metastatic potential of anaplastic thyroid carcinoma cells. J Clin Endocrinol Metab.

[21] Nagarsheth N, Wicha MS, Zou W (2017). Chemokines in the cancer microenvironment and their relevance in cancer immunotherapy. Nat Rev Immunol.

[22] Mantovani A, Savino B, Locati M, Zammataro L, Allavena P, Bonecchi R (2010). The chemokine system in cancer biology and therapy. Cytokine Growth Factor Rev.

[23] Bao G, Clifton M, Hoette TM, Mori K, Deng SX, Qiu A, Viltard M, Williams D, Paragas N, Leete T, Kulkarni R, Li X, Lee B (2010). Iron traffics in circulation bound to a siderocalin (Ngal)-catechol complex. Nat Chem Biol.

[24] Brattain MG, Strobel-Stevens J, Fine D, Webb M, Sarrif AM (1980). Establishment of mouse colonic carcinoma cell lines with different metastatic properties. Cancer Res.

[25] Lee S, Kim JH, Kim JH, Seo JW, Han HS, Lee WH, Mori K, Nakao K, Barasch J, Suk K (2011). Lipocalin-2 Is a chemokine inducer in the central nervous system: role of chemokine ligand 10 (CXCL10) in lipocalin-2-induced cell migration. J Biol Chem.

[26] Jang E, Kim JH, Lee S, Kim JH, Seo JW, Jin M, Lee MG, Jang IS, Lee WH, Suk K (2013). Phenotypic polarization of activated astrocytes: the critical role of lipocalin-2 in the classical inflammatory activation of astrocytes. J Immunol.

[27] Nam Y, Kim JH, Seo M, Kim JH, Jin M, Jeon S, Seo JW, Lee WH, Bing SJ, Jee Y, Lee WK, Park DH, Kook H, Suk K (2014). Lipocalin-2 protein deficiency ameliorates experimental autoimmune encephalomyelitis: the pathogenic role of lipocalin-2 in the central nervous system and peripheral lymphoid tissues. J Biol Chem.

[28] Sickinger S, Maier H, König S, Vallant N, Kofler M, Schumpp P, Schwelberger H, Hermann M, Obrist P, Schneeberger S, Margreiter R, Troppmair J, Pratschke J, Aigner F (2013). Lipocalin-2 as mediator of chemokine expression and granulocyte infiltration during ischemia and reperfusion. Transpl Int.

[29] Lin HH, Liao CJ, Lee YC, Hu KH, Meng HW, Chu ST (2011). Lipocalin-2-induced cytokine production enhances endometrial carcinoma cell survival and migration. Int J Biol Sci.

[30] Jung M, Mertens C, Bauer R, Rehwald C, Brüne B (2017). Lipocalin-2 and iron trafficking in the tumor microenvironment. Pharmacol Res.

[31] Habashy HO, Powe DG, Staka CM, Rakha EA, Ball G, Green AR, Aleskandarany M, Paish EC, Douglas Macmillan R, Nicholson RI, Ellis IO, Gee JM (2010). Transferrin receptor (CD71) is a marker of poor prognosis in breast cancer and can predict response to tamoxifen. Breast Cancer Res Treat.

[32] Brookes MJ, Hughes S, Turner FE, Reynolds G, Sharma N, Ismail T, Berx G, McKie AT, Hotchin N, Anderson GJ, Iqbal T, Tselepis C (2006). Modulation of iron transport proteins in human colorectal carcinogenesis. Gut.

[33] Chekhun VF, Lukyanova NY, Burlaka CA, Bezdenezhnykh NA, Shpyleva SI, Tryndyak VP, Beland FA, Pogribny IP (2013). Iron metabolism disturbances in the MCF-7 human breast cancer cells with acquired resistance to doxorubicin and cisplatin. Int J Oncol.

[34] Chekhun SV, Lukyanova NY, Shvets YV, Burlaka AP, Buchinska LG (2014). Significance of ferritin expression in formation of malignant phenotype of human breast cancer cells. Exp Oncol.

[35] Wang W, Deng Z, Hatcher H, Miller LD, Di X, Tesfay L, Sui G, D'Agostino RB, Torti FM, Torti SV (2014). IRP2 regulates breast tumor growth. Cancer Res.

[36] Reilly PT, Teo WL, Low MJ, Amoyo-Brion AA, Dominguez-Brauer C, Elia AJ, Berger T, Greicius G, Pettersson S, Mak TW (2013). Lipocalin 2 performs contrasting, location-dependent roles in APCmin tumor initiation and progression. Oncogene.

[37] Mauro C, Pacifico F, Lavorgna A, Mellone S, Iannetti A, Acquaviva R, Formisano S, Vito P, Leonardi A (2006). ABIN-1 binds to NEMO/IKKgamma and co-operates with A20 in inhibiting NF-kappaB. J Biol Chem.

